# Perineal Section for Obstruction of Prostatic Urethra

**Published:** 1907-11

**Authors:** 


### PERINEAL SECTION FOR OBSTRUCTION OF PROSTATIC URETHRA.

C. E. Barrett, Ft. Wayne, Ind., sums up the cause and effect of acute retention of urine and considers catheterization more dangerous than operation. He believes bladder drainage through median perineal section the operation of choice. Patients practically moribund recover following improved operative technic with minimum quantity of anesthetic, oxygen inhalation post-operative, Fowler's position, aspiration irrigation with large drain tube and "out of bed" posture second day. Simplicity yet thoroughness in the post-operative treatment is the desideratum.

His case report is a continuation of some cases of like nature reported in the November, 1905, Surgery Gynecology and Obstetrics.

He concludes in his last case reported, that a posterior, gonoccoccal urethritis with its concomitant advancement, i. e., prostatitis, cystitis, Cowperitis, vesiculitis, vasitis and epididymitis,—always remains an infected field. Be it active or passive, mild or virulent, a yearling or a twenty year old, age seems to change the morphology of the coccus not one iota and its host or habitat seems unable to expel it, and unless we can improve on its culture media outside of man that will equal him in its development so that Wright's opsonic indices can be properly taken—until this is accomplished the post gonorrheaic man must be looked upon as a candidate for surgical operative measures for relief sooner or later in life, "More's the pity." (Author's abstract.)

